# Striatal Serotonin Release Signals Reward Value

**DOI:** 10.1523/JNEUROSCI.0602-24.2024

**Published:** 2024-08-08

**Authors:** Mitchell G. Spring, Katherine M. Nautiyal

**Affiliations:** Department of Psychological and Brain Sciences, Dartmouth College, Hanover, New Hampshire 03755

**Keywords:** dorsal striatum, GRAB-5-HT, pavlovian conditioned approach, reward, serotonin, value

## Abstract

Serotonin modulates diverse phenotypes and functions including depressive, aggressive, impulsive, and feeding behaviors, all of which have reward-related components. To date, research has focused on understanding these effects by measuring and manipulating dorsal raphe serotonin neurons and using single-receptor approaches. These studies have led to a better understanding of the heterogeneity of serotonin actions on behavior; however, they leave open many questions about the timing and location of serotonin's actions modulating the neural circuits that drive these behaviors. Recent advances in genetically encoded fluorescent biosensors, including the GPCR activation-based sensor for serotonin (GRAB-5-HT), enable the measurement of serotonin release in mice on a timescale compatible with a single rewarding event without corelease confounds. Given substantial evidence from slice electrophysiology experiments showing that serotonin influences neural activity of the striatal circuitry, and the known role of the dorsal medial striatal (DMS) in reward-directed behavior, we focused on understanding the parameters and timing that govern serotonin release in the DMS in the context of reward consumption, external reward value, internal state, and cued reward. Overall, we found that serotonin release is associated with each of these and encodes reward anticipation, value, approach, and consumption in the DMS.

## Significance Statement

Serotonin influences many reward-related phenotypes including those dysregulated in a number of psychiatric and neurological disorders. A large amount of research has focused on the role of dopamine in mediating striatal reward circuits, though we know that serotonin has the potential to modulate striatal circuitry and reward behaviors. Using the recently developed serotonin biosensor, GRAB-5-HT, we were able to resolve the timescale and components of reward that are encoded by striatal serotonin. Our results show that serotonin is released in anticipation of a reward and also in response to a cue that predicts a reward, and the long duration signal is graded by extrinsic and subjective value.

## Introduction

While dopamine has most commonly been implicated in the neural mechanisms that underlie motivation and goal-directed behavior, there is a substantial amount of research that implicates serotonin in value-driven action selection. Brain-wide manipulations show that serotonin promotes goal-directed behavior over habitual responding in rodents and humans (Worbe et al., 2015; Ohmura et al., 2021). These effects may be mediated via serotonin's contribution to encoding the representation of reward value; however, there are heterogeneous results from investigations probing this idea. While serotonin depletion is associated with reduced neural responsivity to rewards (Seymour et al., 2012), increased synaptic serotonin does not alter hedonic liking (Treit and Berridge, 1990; Caras et al., 2008) or incentive wanting (Nonkes et al., 2014). At the receptor level, there is strong evidence that a number of serotonin receptor subtypes, including the 1A, 1B, 2A, 2C, and 3 receptors, influence reward-related behavior, possibly through reward valuation (Hayes and Greenshaw, 2011; Nautiyal and Hen, 2017; Desrochers et al., 2021; Desrochers and Nautiyal, 2022). Though these gross manipulations implicate serotonin in reward-related behaviors, it is unclear how serotonergic signaling fits into the well-known neural mechanisms supporting reward-based decision-making or the encoding of reward value.

Studies investigating the role of serotonin in reward have primarily examined the somatic activity of neurons in the dorsal raphe nucleus (DRN) using electrophysiology or fiber photometry. Some studies suggest that DRN activity increases following the presentation of unexpected reward and also ramps up during expectation of reward (Cohen et al., 2015; Li et al., 2016; Zhong et al., 2017). DRN neurons also display prediction error-like signaling, particularly under conditions of uncertainty, that adapts to learning on a different timescale than dopamine prediction error (Matias et al., 2017). Other data show that DRN neurons respond to innate and conditioned rewards as well as prospective value (Nakamura et al., 2008; Bromberg-Martin et al., 2010; Zhou et al., 2015; Zhong et al., 2017). These results are consistent with the diversity of behavioral effects induced by serotonin manipulation, such as enabling goal-directed action (Ohmura et al., 2021), using cue expectations to update behavior (Walker et al., 2022), and persisting in reward seeking (K. W. Miyazaki et al., 2014). Together, these varied responses to reward and effects of DRN manipulation have led to numerous theories regarding the DRN's function, including representing subjective well-being, uncertainty and behavioral flexibility, and/or the value of persistence (M. Luo et al., 2016; Doya et al., 2021; Grossman et al., 2022; Q. Luo et al., 2023).

A caveat in these approaches is that DRN neurons show heterogeneity in cotransmission, projection targets, presynaptic neuromodulatory mechanisms, and volume transmission, making DRN somatic activity data insufficient to understand the role of serotonin in reward-related behavior. There are also differential responses of DRN serotonin neurons based on their projection targets. DRN neurons respond to emotionally salient stimuli regardless of the valence but display projection-specific biases in encoding appetitive versus aversive stimuli (Paquelet et al., 2022). For example, while cortical-projecting neurons are activated in response to reward, amygdala-projecting neurons are activated by both rewarding and aversive stimuli (Ren et al., 2018; Paquelet et al., 2022). While these differences could be addressed with projection-specific monitoring of cellular activity, still DRN neurons are molecularly heterogeneous and many corelease glutamate, making the somatic activity of DRN neurons an unreliable readout of serotonin function (Ren et al., 2019; Wang et al., 2019; Okaty et al., 2020). Additionally, serotonin neurons have presynaptic modulatory mechanisms which allow for regulation of serotonin signaling independent of somatic activity. Finally, given that serotonin can signal via volume transmission, even the activity of a homogeneous population of projection-specific serotonin neurons may not reflect functional serotonin signaling on a time-scale consistent with encoding reward associations (Agnati et al., 1995). Therefore monitoring only the somatic activity of these neurons is insufficient to understand the role of serotonin in reward-related behaviors, and looking at the release of serotonin in relevant brain regions is important for a more complete understanding serotonin's involvement in reward processing.

Reward based decision-making relies on the striatum, which receives substantial serotonin input and expresses most serotonin receptor subtypes, in addition to well-described canonical dopamine-based circuitry (Nair et al., 2020). A number of in vitro studies show that serotonin specifically shapes the neural activity of the dorsomedial striatum (DMS), a region necessary for action selection and learning about action–outcome associations. Serotonin gates the release of other neurotransmitters, including dopamine and glutamate, into the dorsal striatum while simultaneously modulating striatal microcircuits and projection populations. Evidence from slice electrophysiology shows that serotonin causes long-term depression of glutamatergic cortical inputs to medium spiny neurons (MSNs; Mathur et al., 2011), excites both cholinergic (Virk et al., 2016) and fast-spiking interneurons (Blomeley and Bracci, 2009; Muñoz-Manchado et al., 2016), and can inhibit collateral inhibition between MSNs (Pommer et al., 2021; Burke and Alvarez, 2022). Additionally, serotonin can regulate the release of dopamine directly to influence conventional signaling mechanisms of the DMS (Nair et al., 2020). Given that there is strong evidence to support serotonin modulation of striatal circuitry, it is surprising that few studies have investigated this in vivo. There are many known mechanisms through which serotonin could influence striatal neurophysiology in vitro which makes it critical to determine the temporal characteristics of serotonin release in the context of rewards, reward-predictive cues, and motivated behavior. Past work has been limited in this respect because of the low temporal resolution of microdialysis, the primary technique previously available for monitoring 5-HT release in vivo. This characterization is an important step toward understanding how serotonin contributes to information processing in the DMS during goal-directed action.

The recent availability of serotonin biosensors such as G-protein receptor activation-based (GRAB)-5-HT allows the measurement of serotonin release on a timescale that allows trial-by-trial analysis to examine the timing of the release in the context of reward (Wan et al., 2021; Deng et al., 2024). We focus on the DMS based on its known role in mediating reward-related behaviors and strong evidence that serotonin modulates DMS neural activity. This approach enables the identification of the aspects of reward processing that are encoded by serotonin without the confounds of glutamate corelease or unknown projection targets. We focused on measuring the release of serotonin in the DMS during reward consumption of different reward values and during varied internal states, as well as in a pavlovian appetitive paradigm to understand how serotonin encodes different features of reward. Our data show that serotonin release in the DMS corresponds to features of reward anticipation, approach, and consumption. Specifically, serotonin is released into the DMS in anticipation of a reward, beginning to rise ∼2 s before consumption corresponding to anticipatory motivation. This anticipatory release is also seen during a cue that predicts a reward in a pavlovian conditioning experiment. Additionally, the amount of serotonin release during reward consumption correlates with external reward value and is also influenced by subjective value which we varied by changing internal state. Overall, our results show that serotonin is a signal of anticipated outcome value in the DMS and can serve to modulate reward-related behavior.

## Materials and Methods

### Animals

All mice were bred in the Center for Comparative Medicine and Research at Dartmouth College and weaned at postnatal day 21 into cages of 2–4 same-sex littermates until surgical fiber-optic implantation. The mouse colony was established from C57BL/6J mice (Strain 000664, Jackson Laboratory). Mice were maintained on a 12 h light/dark cycle on *ad libitum* food and water until experimental testing began at 15–21 weeks of age. During experimental training and testing, food was provided daily, to maintain a targeted body weight of 80–90% of their free-feeding weight, with *ad libitum* access to water. Water was withheld once for 24 h prior to testing the effect of internal thirst state. Gustometer experiments for reward consumption and reward value experiments were performed on *N* = 11 mice (5 male, 6 female). Pavlovian approach experiments were performed on *N* = 16 naive mice (10 male, 6 female). Experiments testing internal state using water restriction were performed on *N* = 15 mice (9 male, 6 female) which included *N* = 7 mice from the pavlovian approach experiment and an additional *N* = 8 mice (4 male, 4 female). These latter *N* = 8 mice were also used in the satiety-induced devaluation study with an additional *N* = 8 mice (5 males, 3 females). Following fiber-optic implantation, mice were singly housed and provided with environmental enrichment.

### Behavior

#### Gustometer

Mice were trained to retrieve rewards in a Davis Rig Gustometer (MED Associates). Water and varied concentrations of evaporated milk (Carnation brand) reward were provided, and licking was measured via capacitance-based lick sensing. Training began ∼4 weeks following GRAB-5-HT viral infusion/fiber implantation surgery. Initial training sessions consisted of 15 min uninterrupted access to a single bottle of 100% evaporated milk. These sessions continued until mice displayed licking to the reward. Following initial training, mice were trained in 40 min sessions with intermittent access to 100% evaporated milk, with access gated by a shutter door. Following the initiation of licking on each trial, the shutter door remained open for an additional 5 s before closing. Following each trial, the door remained closed for 7.5 s before opening again to initiate the next trial. If the mouse did not lick within 60 s of the door's opening, the trial was considered an omission, and the door closed for 7.5 s. Following this basic training, mice were tested in two-bottle (water vs 100% evaporated milk) and six-bottle (0, 20, 40, 60, 80, and 100% evaporated milk) paradigms, in which one bottle was randomly presented at each trial. For these paradigms, access to each concentration was extended to 10 s following the first lick, the intertrial interval (ITI) was 15 s, and the maximum wait was shortened to 30 s. Additionally, up to two re-presentations were offered following an omission, in which the same concentration would be reoffered following the standard interpresentation interval. In the six-bottle task, the number of licks during each presentation was the primary measure analyzed.

In the water versus 100% milk task, a composite preference score was used to compare relative preference for milk between satiety states. Relative preference for milk over water was quantified using a composite metric calculated based on the average of three components: (1) median number of licks, (2) interlick interval, and (3) latency to consume for each solution [(1) Lick-based preference = (Milk − Water) / (Milk + Water); (2) ILI-based preference = (Water − Milk) / (Range of ILIs for mouse); (3) Latency-based preference = (Water − Milk) / 30]. Animals were initially excluded from signal analysis on the basis of having a preference score during water restriction that reflected a less than twofold decrease from their score during food restriction, reflecting that these mice were potentially not thirsty enough to induce an increase in the subjective value of water. Mice in the satiety devaluation study were given access to banana- and chocolate-flavored (Nesquik) 50% evaporated milk to assess baseline consumption and GRAB-5-HT signal. This paradigm was identical to that of standard two-bottle paradigm described above. On the following day, they were placed in the gustometer and given unrestricted (i.e., the shutter never closed) access to either banana or chocolate (counterbalanced across animals and based on initial preference) for 1 h. Then mice were run in the standard two-bottle paradigm while consumption of the two flavors and GRAB-5-HT signal were measured. Preference was calculated for both baseline and prefed days according to the metric described above, with positive values indicating preference for the devalued flavor.

#### Pavlovian conditioning

Mice were run in one of four Bussey-Saksida operant touch-screen chambers (Lafayette Instrument). These are trapezoidal chambers with stainless steel floors and black walls, enclosed in sound-attenuating box. Evaporated milk reward was delivered via a peristaltic pump, and retrieval was detected via infrared beam breaks. LED houselights and speakers were mounted above the chamber. Delivery of evaporated milk (US) was predicted by the presentation of an 8 s auditory stimulus (CS+; white noise or pure 5 kHz tone, counterbalanced across animals). Delivery of the unpaired stimulus was interspersed among CS+/US pairings and served as a CS−. CS delivery occurred with an average ITI of 45 s. A single conditioning session comprised 15 CS+ and 15 CS− presentations. Mice received one session per day for 10 d. Movement within the chamber was tracked via three infrared (IR) beams: one on the side of the box opposite the reward receptacle, one in front of the reward receptacle, and one inside the reward receptacle itself. Time spent near the reward receptacle was quantified by tracking interruptions of the second IR beam and binarized in 100 ms bins for each trial and aligned to CS+ or CS− onset. The magnitude of reward receptacle approach was calculated by averaging approach across all trials of a given cue presentation for each mouse within each session and then normalizing the resultant trace relative to an 8 s pre-CS/ITI baseline. The mean of the trace was also calculated during the cue and reward periods and used as a covariate in the ANCOVA analysis of the GRAB-5-HT.

### GRAB-5-HT photometry

#### Surgery

Mice were anesthetized under isoflurane anesthesia and head-fixed for stereotaxic infusion of the GRAB-5-HT3.6 biosensor and implantation of an optic fiber. A viral vector expressing the GRAB-5-HT biosensor (AAV9-hSyn-GRAB3.6; WZ Biosciences) was injected into the DMS [AP: + 0.8 mm; ML: + 1.6 mm; DV: −3.0 mm from skull] with an injection volume of 0.5 μl infused over 10 min at a titer = 1.5 × 10^13^ molecules/ml]. Next, an optic fiber (3.5 mm length, 400 μm core/430 μm outer diameter, 0.48 numerical aperture, flat tip; Doric) was implanted at the same site just over the injection [AP: + 0.8 mm; ML: + 1.6 mm; DV: −2.8 mm from skull]. The fiber was affixed to the skull surface using dental cement (Metabond) and skull screws. Mice were treated with the anti-inflammatory drug ketoprofen (100 mg/kg) on the day of surgery and for 4 d following surgery to reduce inflammation and postoperative pain.

#### Placement

**Figure 1.**
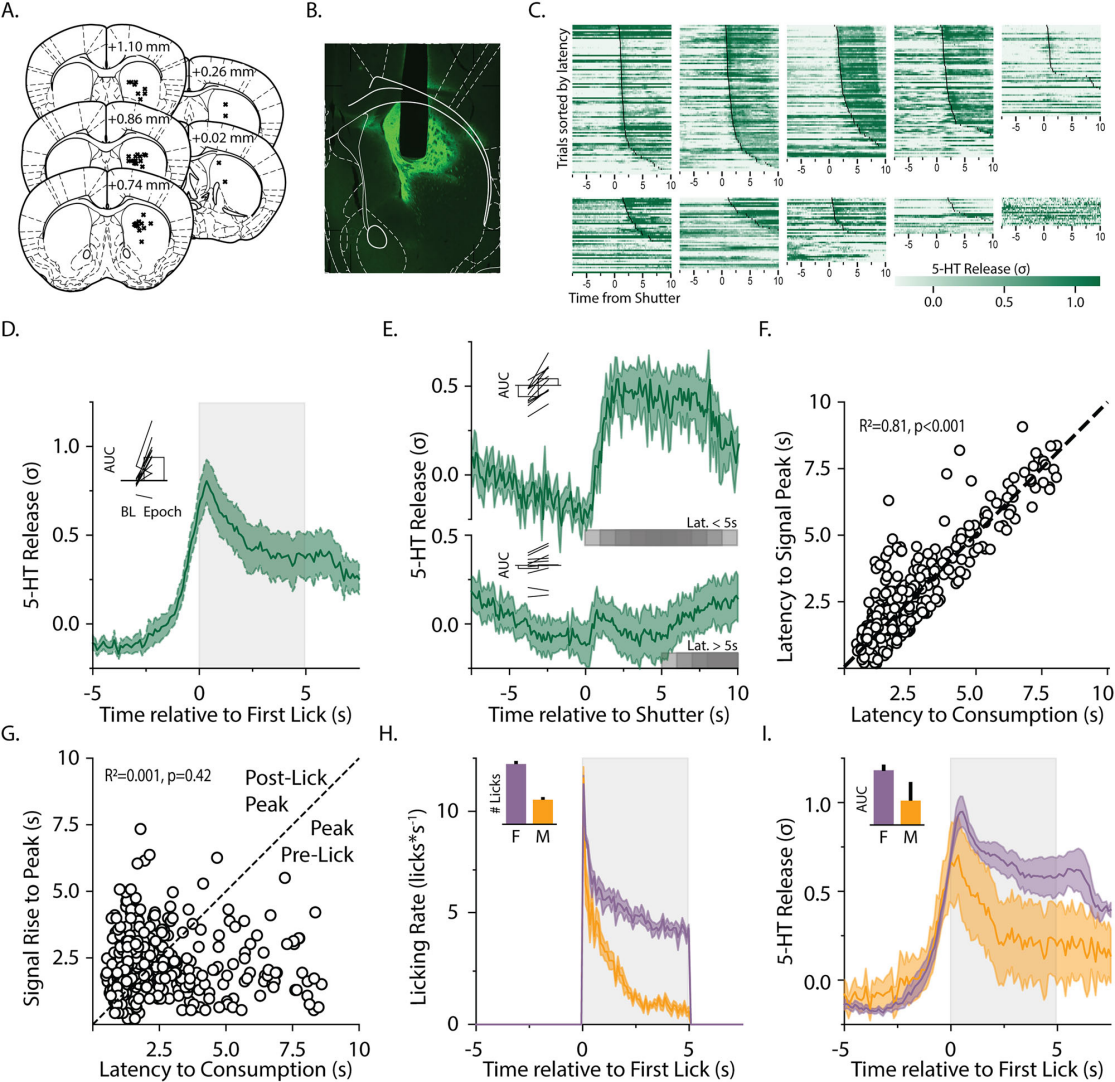
Serotonin release in the DMS precedes reward consumption. ***A***, Fiber placements from mice from GRAB-5-HT recordings are shown by an x for each animal included in the analysis. All markers are displayed on one hemisphere on the coronal brain plates for illustrative purposes, but fiber placements were counterbalanced across hemispheres. ***B***, A representative image shows the fiber track and GRAB-5-HT expression. ***C***, The *z*-scored GRAB-5-HT fluorescence signal is shown for 10 mice with each row representing a single trial aligned to the shutter opening (triangle) and sorted by latency to first lick. The onset of licking in each trial is indicated by a black dot overlaying the signal. ***D***, The trial-averaged *z*-scored GRAB-5-HT fluorescence signal is shown averaged over all trials across all mice, aligned to the first lick of each trial. The 5 s period of reward availability is shown in gray. Inset shows individual mice (lines) and group averages for the AUCs for baseline and reward period epochs. ***E***, The trial-averaged *z*-scored GRAB 5-HT fluorescence signal is shown averaged over trials separated by latencies to lick of shorter or longer than 5 s, and aligned to shutter opening. Gray shading indicates where licking may be occurring depending on the given latency greater than 5 s for each trial. Inset shows individual mice and group averages for AUCs of baseline and postshutter periods for the short and long latency trials. ***F***, For every trial, the latency to begin licking is plotted against the timing of the signal peak following shutter open. ***G***, For each trial for all animals, the latency to begin licking is plotted against the rise time-to-postlick peak of the signal during that trial, showing signal rise times tend to be shorter than behavioral latency. Sex differences in licking (***H***) and GRAB-5-HT signal (***I***) are shown between males and females with insets displaying the area under the curve during the licking epoch (gray shading).

Brain tissue was fixed via intracardiac perfusion with 4% PFA prior to the removal of the cranium and placement in 4% PFA. Twenty-four hours later, the brain was removed from the cranium and placed in fresh PFA for 24 h before being placed in 30% sucrose prior to freezing at −80C. Frozen tissue was sectioned at 35 μM on a cryostat (Leica) and stored in 1× PBS. Tissue sections were labeled with DAPI prior to wet mounting on slides and were visualized at 4× magnification in a fluorescent microscope. Fiber placements were localized and shown in Figure 1*A*,*B*. Placement was unable to be confirmed for one mouse that was originally used in the satiety-induced devaluation study (presented in Fig. 4); this mouse was excluded from analysis and does not contribute to the *N* reported above.

#### Data collection

GRAB-5-HT recording began 4 weeks following viral infusion and fiber implantation. Simultaneous recording of GRAB-5-HT fluorescence was accomplished using two separate wavelengths of light at 465 and 405 nm provided by two single wavelength LEDs, driven by Doric Neuroscience Studio software. Both wavelengths were routed through a dichroic mirror (4-port fluorescence minicube, Doric) into a low-autofluorescence patchcord (400 μm core, 0.48 numerical aperture). This fiber was secured to the optic fiber implanted in the animal using either a ceramic mating sleeve (Doric) or a quick-release interconnect (ADAL3 Thorlabs). This fiber carried both the excitation and emission fluorescence, which were separated by a dichroic mirror that delivered the fluorescence to a Newport Visible Femtowatt photoreceiver (Doric; delivered by 600 μm core/630 μm outer diameter, 0.48 numerical aperture patch cord, Doric). Doric Neuroscience Studio software (Doric) was used to collect the fluorescent recordings at 1,017.2 Hz, apply lock-in demodulation to separate the excitation and isosbestic fluorescences, and simultaneously record behavioral events via TTL input from behavioral apparatus in the same data file.

#### Signal preprocessing

Signal preprocessing included low-pass filtering, photobleaching detrending, motion correction, and normalization, as described by Simpson et al. (2024). Prior to analysis, a 5 Hz Butterworth low-pass filter was applied to the signal, and it was down-sampled to 10 Hz. Subsequently, a two-phase exponential decay curve was fitted to, and subtracted from, the GRAB emission and isosbestic signals (a separate decay curve being used for each signal) to remove the influence of autofluorescence and indicator photobleaching. Motion correction of the GRAB-5-HT signal was performed in multiple steps. First, the detrended isosbestic signal was regressed against the detrended GRAB-5-HT signal to estimate the component of signal fluctuation accounted for by motion. Second, the coefficients of this regression were used with the detrended isosbestic to estimate motion in each sampling frame. Third and finally, this motion estimate was subtracted from the GRAB-5-HT signal. The resultant signal was normalized through conversion to robust median *Z* scores: 0.6475 * (*x_i_* − *x̃*) / (MAD), where *x_i_* is an individual datapoint, *x̃* is the median signal during a reference period, and MAD is the median absolute deviation of the signal during that period; the quantity is scaled to the standard mean-based *z*-distribution through multiplication by the constant, 0.6475. The entire session was used as the reference period for data collected during self-paced consumption in the lickometer, where changes in signal preceded the behavior used for alignment, and normalization was performed prior to alignment with behavior. For data from the pavlovian conditioning task, data were first aligned by trials, and then each trace was normalized against the median and MAD of an 8 s pretrial baseline.

### Analysis

#### GRAB-5-HT signal processing

Signal alignment to behavior was performed using the TTLs generated by the behavioral apparatus. In the Davis Rig Gustometer, a TTL was sent to the Doric Photometry Console at the time of the first lick in each trial. The time of the shutter's opening in each trial was determined by subtracting the latency to lick for each trial, recorded in behavioral output files by the lickometer, from the timestamp of the TTL associated with the same trial, matched based on index. One animal was excluded from all shutter-aligned analyses (shown in Fig. 1*C* and *E*) due to a mismatch between the number of TTLs received by the Photometry Console and the number of trials recorded in the behavioral file. In the Bussey-Saksida boxes, TTLs were generated at the beginning of the session, at the onset of tone delivery (for either CS), the delivery of reward, and during any nosepoke into the reward port. Average event-aligned signals were then calculated for each mouse and used for analyses and graphing. For quantification of behaviorally aligned signal, area under the curve was approximated using trapezoidal summation.

To calculate shutter-aligned signal peaks and troughs in the gustometer (for the analysis shown in Fig. 1*F,G*), periconsumption peak and prelick trough were identified by applying a fifth-order smooth to the shutter-aligned GRAB-5-HT signal (in order to capture only the overall signal trend surrounding the shutter), computing the *z*-normalized first derivative of the smoothed signal, and applying the following procedure: First, identify all points in the normalized derivative above a dynamic threshold value (initially set to −0.75). If all points after 1 s prior to the lick are above that threshold, increment the threshold by 0.1, and repeat until the signal can be separated into points above and below the threshold. Second, beginning 1 s prior to the lick, identify the first point at which the signal falls below the selected threshold. The periconsumption peak is defined as immediately preceding the point at which the signal falls. Third, identify the final preconsumption point where the normalized derivative is below the threshold. This is the preconsumption trough.

#### Transient analysis

Identification of GRAB-5-HT signal events was adapted from Ca^2+^ transient identification procedure used for GCaMP fluorescence (Moda-Sava et al., 2019). Briefly, periods of signal that exceeded 0.5 MAD (based on the median of the entire session) were identified as candidate events. The end point of each event was defined as the point at which the signal fell below 0.5 MAD. The start point was defined as the point at which the *z*-normalized first derivative of the signal exceeded 1 MAD in the signal's rise to threshold. Events that occurred within 0.5 s of a prior event were linked to that event. Events that lasted <0.5 s were filtered out. The magnitude of each event was defined as the mean signal during the transient, so that signal duration could be considered separately. To determine whether transients were more likely to occur during licking, random offsets were iteratively introduced between the neural signal and lick times (up to 20 min in either direction, with values at the end of a range wrapping around to the other side following the offset; 2,000 repetitions).

#### Statistics

ANOVA was used for all omnibus analyses. Post hoc testing was performed using simple contrasts, with the Holm method being used to correct for multiple comparisons. *t* tests were used for simple comparisons where valid, with the exception being for the median and magnitude data for the transient analysis in which the Wilcoxon rank sums and Kruskal–Wallis tests were used due to the non-normal distribution of transients. The mediation analysis was performed using structural equation modeling. We estimated 95% confidence intervals for reported coefficients via bootstrap resampling (2,000 iterations). For analysis of pavlovian conditioned approach data, behavioral approach to the reward receptacle during different trial periods, cues, and training time points were compared using a 2 (Epoch: Cue vs Reward period) × 2 (CS-type: CS+ vs CS− trial) × 2 (Training: Early vs Late Training) mixed ANOVA. This analysis was repeated for the GRAB-5-HT signal using behavioral approach as a covariate in an identically structured (Epoch × CS−Type × Training) ANCOVA. All statistics were performed in *R* with *α* = 0.05 for all tests.

## Results

### DMS serotonin release precedes reward consumption

To characterize the timing of serotonin release in the DMS in the context of rewards, we analyzed the fluorescence emitted from a GRAB-5-HT biosensor during reward consumption measured by a capacitance-based lick sensing gustometer. Normalized fluorescence was first aligned to the beginning of each trial marked by the availability of the reward and sorted by latency to lick the reward spout. The timing of the serotonin release during each trial corresponded with the onset of licking for reward, as seen clearly when trials were sorted by the latency to lick for the reward (Fig. 1*C*). Next, aligning the GRAB-5-HT fluorescence signal for each trial to the time of the first lick illustrates that the serotonin release begins prior to the onset of reward consumption, beginning to ramp up ∼2 s before the first lick (Fig. 1*D*; median rise time from prelick trough to lick-associated peak: 1.9 s). Serotonin levels remained significantly elevated during the 5 s of licking during reward availability (*t*_(10)_ = 4.29; *p* = 0.0016).

There was a small but potentially significant increase in serotonin associated with the shutter opening, which was audible and signaled reward availability at the beginning of each trial (*t*_(10)_ = 2.586; *p* = 0.029). This was analyzed using trials with latencies to initiate licking that were longer than 5 s, which included 207 out of 671 total trials across 10 animals (Fig. 1*E*). Comparing these trials to short latency lick trials (which had lick latencies of shorter than 5 s), there was a significant effect of trial latency on the serotonin response following the shutter (period × trial latency interaction: *F*_(1,9)_ = 17.367; *p* = 0.0024). This suggests that serotonin is likely responsive to the auditory stimulus or reward availability, though it much more strongly encodes reward consumption. This was further demonstrated by the significant correlation between the latency to initiate licking for the reward and the latency to the peak of the GRAB-5-HT signal, showing strong alignment with licking, regardless of how long they took to lick on any trial (Fig. 1*F*; *R*^2 ^= 0.81; *p* < 0.0001). Furthermore, we also analyzed the GRAB-5-HT signal dynamics in relation to consumption latency on individual trials to determine if there was a ramping of serotonin release prior to consumption. The signal rise time (between the signal peak and the preceding local minima) was not significantly correlated with the latency to lick (Fig. 1*G*; *R*^2 ^= 0.0015; *p* = 0.421). Overall these analyses indicate that serotonin release begins in anticipation of reward consumption and peaks around the onset of consumption.

Analysis of sex differences showed that males and females consumed significantly different amounts of sucrose reward as measured by lick rates over each trial (*F*_(1,9)_ = 12.64; *p* = 0.0006). Though they both exhibited similar lick rates at the beginning of trials, the rates of males fell sharply while females maintained higher lick rates throughout the trial (Fig. 1*H*). Interestingly, the sex difference in consumption behavior was not reflected in differences in GRAB-5-HT signal. While there was some trend for females to have a prolonged elevation in the GRAB-5-HT signal during reward consumption (Fig. 1*I*), there was no statistically significant difference in GRAB-5-HT signals between males and females over the 5 s licking period (*F*_(1,9)_ = 1.90; *p* = 0.201).

### Serotonin levels encode reward value in the DMS

**Figure 2.**
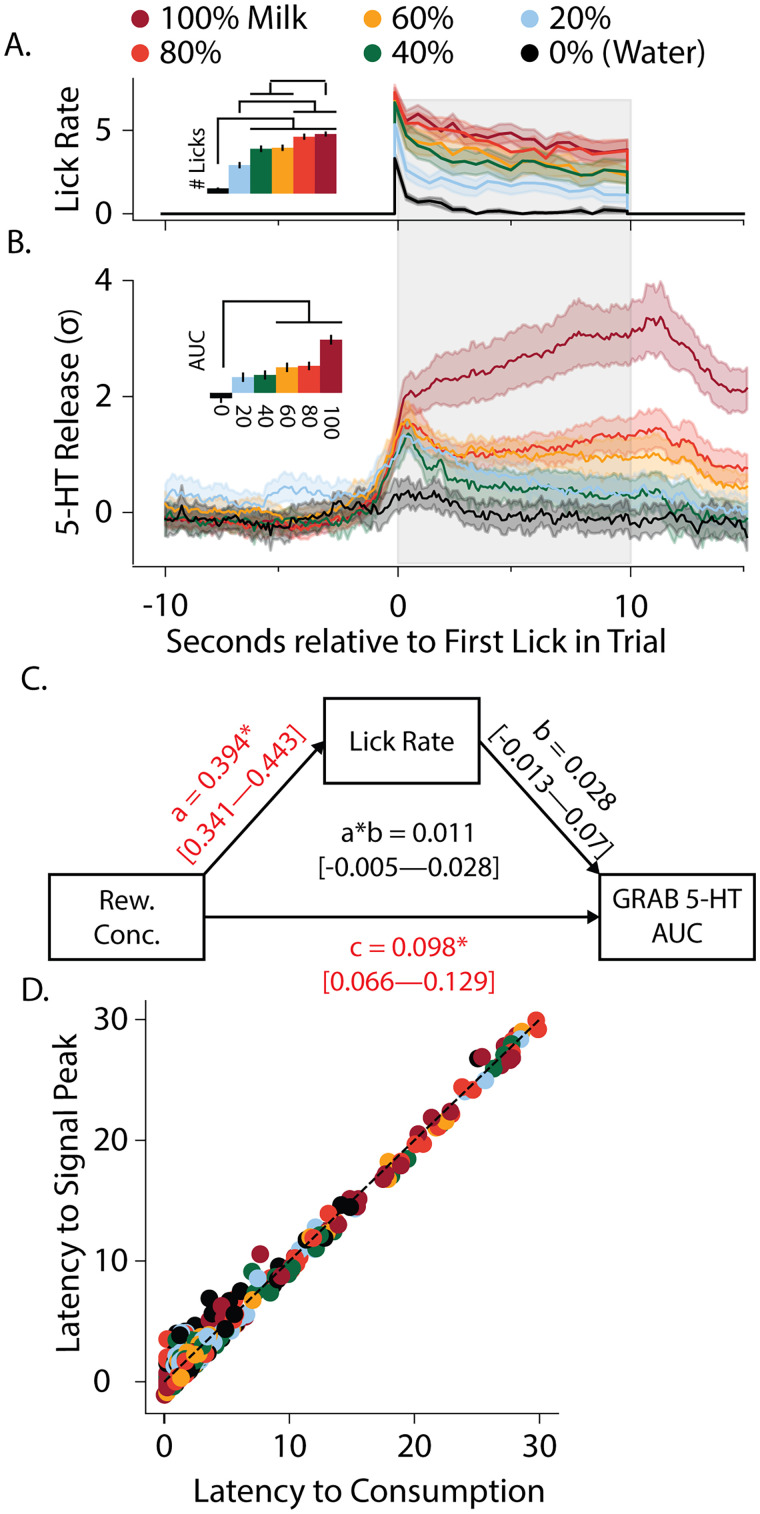
Serotonin levels in the DMS encode reward value. ***A***, Behaviorally, lick rate increases with increasing reward concentration. The bar graph inset shows the group average of number of licks directed toward each concentration over all trials in all animals. ***B***, The lick aligned GRAB-5-HT signal is shown for each reward concentration with the bar graph inset showing the group averages of the area under the curve during the 10 s consumption period for each concentration, ***C***, A mediation analysis found independent, significant influences of reward concentration on both lick rate and GRAB-5-HT signal (significance indicated in red) and no evidence that lick rate could explain the correlation between reward concentration and serotonin release. ***D***, The latency to consume reward (in seconds) is plotted against the latency to the peak signal (in seconds) across all concentrations of reward from all trials.

To investigate how serotonin release varies with reward value, we presented six different concentrations of reward and measured licking to each reward concentration and the associated GRAB-5-HT signal. Overall, higher value rewards were associated with higher serotonin levels. Behaviorally, mice displayed more licking for higher reward concentrations as expected (Fig. 2*A*; *F*_(5,45)_ = 10.55; *p* = 0.001). Interestingly, there was also a significant main effect of reward value on serotonin release (Fig. 2*B*; *F*_(5,45)_ = 18.06.15; *p* < 0.001), with 60, 80, and 100% reward concentrations being associated with significantly higher serotonin levels compared with water (0%) reward (*Ψ*_(9)_ = 3.87; *p*_holm _= 0.0038 for 60%; *Ψ*_(9)_ = 5.32; *p*_holm _= 0.0005 for 80%; *Ψ*_(9)_ = 5.45; *p*_holm _= 0.0004 for 100%). Here again, the latency to consume the reward was associated with the latency to the signal peak (Fig. 2*D*; ßConsume = 0.865; *p* < 0.001), but it was not significantly influenced by concentration (ßConcentration = −1.19, *p* = 0.061; ßConsume × Concentration = 0.03, *p* = 0.886). To determine if the increased serotonin was due to differences in reward value independent of the increased licking, we performed a mediation analysis using structural equation modeling testing lick rate as a mediator variable (Fig. 2*C*). We found that while reward concentration was significantly correlated with both lick rate and serotonin levels (95% CI = 0.341–0.443 for lick rate and 95% CI = 0.066–0.129 for GRAB-5-HT AUC), the relationship of reward concentration on serotonin levels was independent of lick rate. Specifically, there was no direct effect of lick rate on serotonin levels (95% CI = −0.013 to 0.07), and the relationship between reward value and serotonin signal was not mediated by lick rate (95% CI = −0.005 to 0.028). This shows that the increased serotonin levels to higher reward values were not due to the increased licking to higher reward concentrations and suggests the ability for serotonin in the DMS to encode value.

Additionally, analysis of the GRAB-5-HT signal during the 0% reward concentration (water) presentations shows that the serotonin signal is not increased during water consumption, illustrating that serotonin release is associated with reward rather than locomotion or fluid consumption aspects of this paradigm. Specifically, the GRAB-5-HT fluorescence aligned to the onset of licking for water was not significantly different from the 10 s baseline preceding licking (*t*_(9)_ = 0.592, *p* = 0.569), which includes locomotive and initial licking behavior similar to that seen for all presentations of reward concentrations.

**Figure 3.**
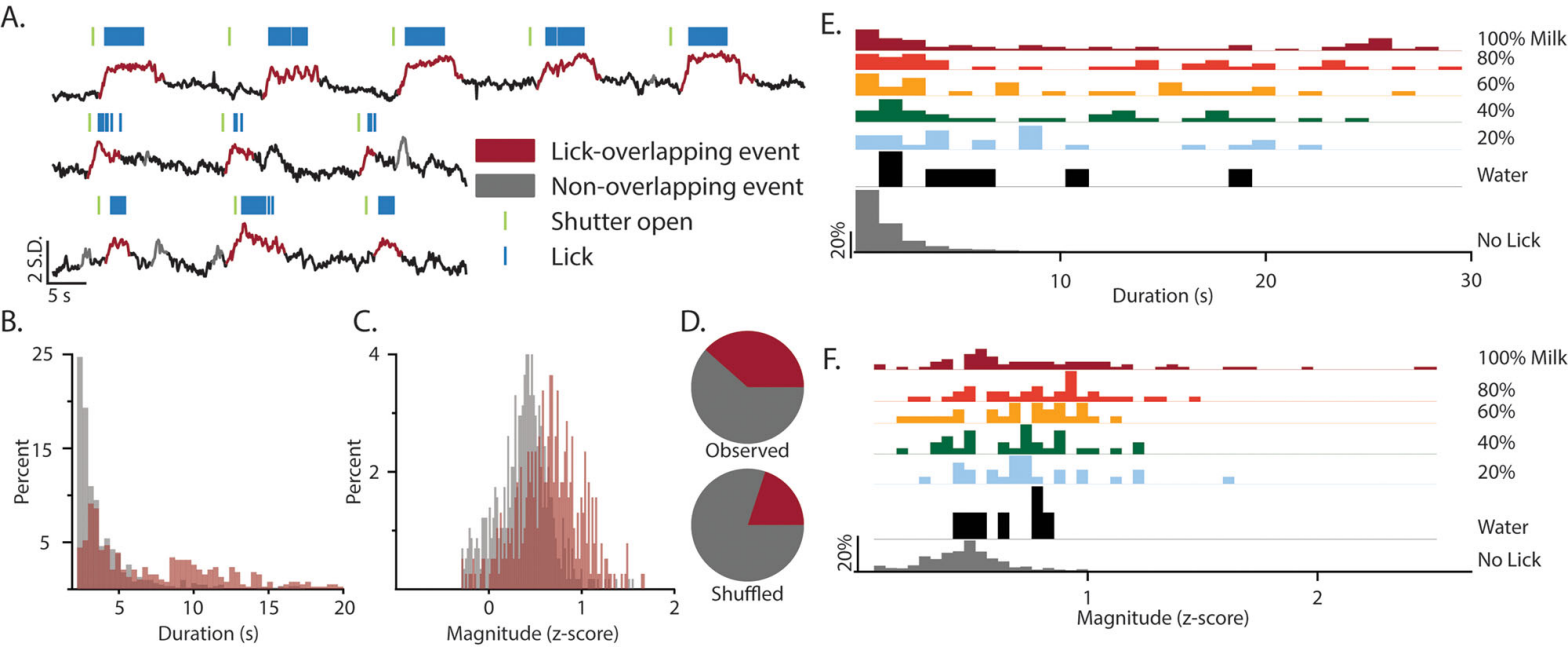
Transient-based analysis of DMS serotonin signal identifies serotonin signals are preferentially related to reward. ***A***, Example traces demonstrating transient identification and categorization. Algorithmically identified transients were categorized as lick-overlapping (red) or not (gray). Histograms display the distribution of transient durations (***B***) and magnitude (***C***) for lick-overlapping (red) and nonoverlapping (gray) transients. ***D***, The proportion of transients that overlap licking is shown for observed (top) and shuffled (bottom) data, showing a significantly higher than chance overlap of 5-HT transients with licking. The distribution of the duration (***E***) and magnitude (***F***) of 5-HT transients that occurred during consumption of each concentration of reward (red, orange, yellow, green, blue) are shown in comparison with the transients that occurred during water consumption (black) or in the absence of any consumption (gray) while both metrics are positively skewed for transients that overlap multiple concentrations of milk.

To analyze the extent to which serotonin release in the DMS was preferentially related to reward, we identified transients in the GRAB-5-HT fluorescent signal independent of any behavioral events and then subsequently categorized each transient based on the licking behavior (Fig. 3*A*). Quantification of the duration and magnitude of the transients showed that GRAB-5-HT transients were significantly more robust if they occurred during reward licking bouts. Specifically, lick-overlapping GRAB-5-HT transients were both longer (Fig. 3*B*; *w* = 181,430; *p* < 0.001) and larger (Fig. 3*C*; *w* = 177,645; *p* < 0.001) than transients that occurred in the absence of reward consumption. This showed that the number of transients which overlapped with reward consumption was significantly greater than predicted by the chance distribution (Fig. 3*D*; *χ*^2^_(1)_ = 247.46; *p* < 0.0001). This suggests that serotonin is preferentially released into the DMS during reward compared with other nonreward events during the recording session.

**Figure 4.**
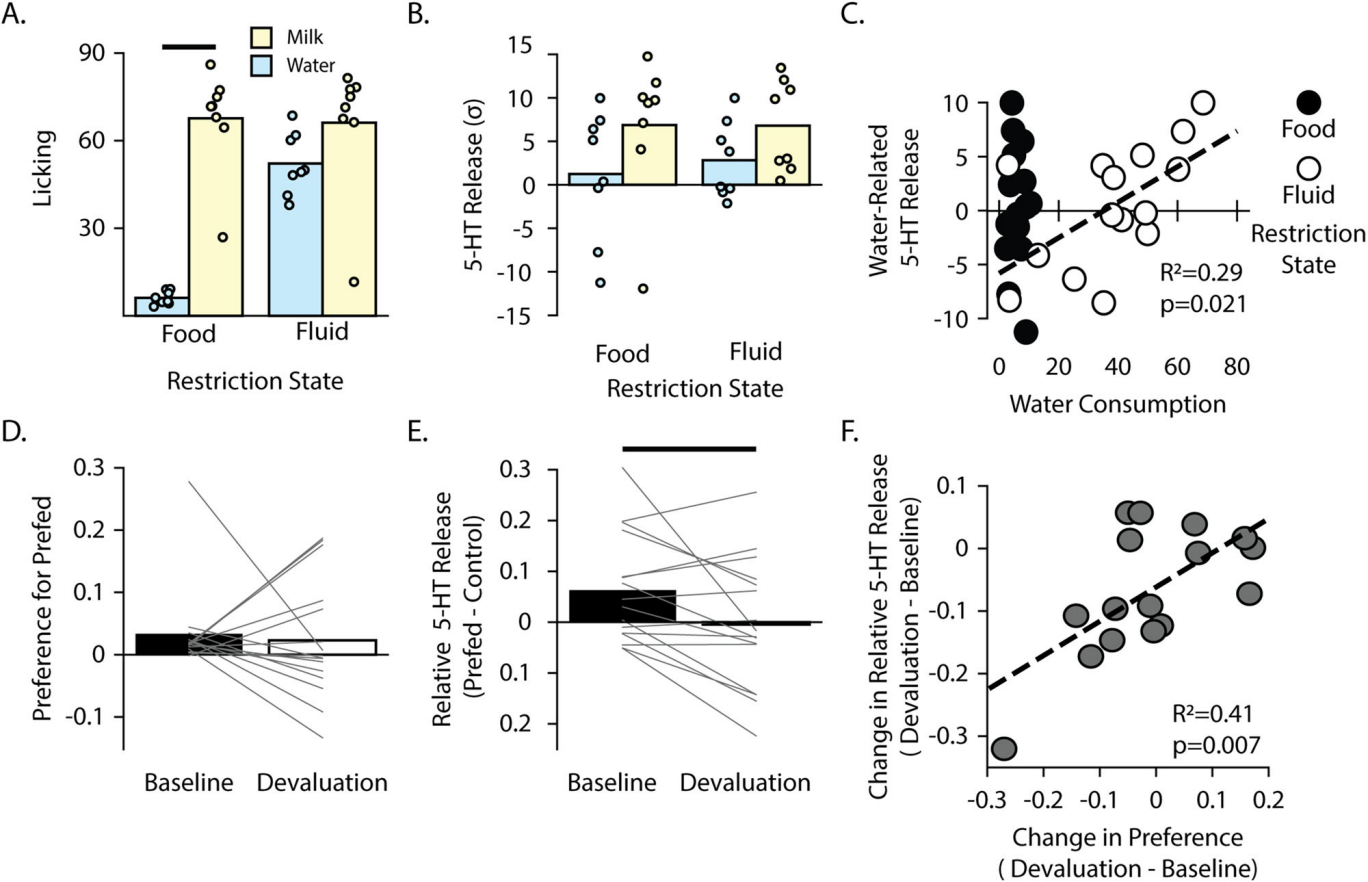
Internal state influences reward-related serotonin release in the DMS. ***A***, Consumption of water and 100% milk under water-restricted and food-restricted conditions. ***B***, The area under the curve of the GRAB-5-HT signal is shown during consumption of water or evaporated milk for mice under water-restricted or food-restricted conditions. ***C***, The amount of water consumed is plotted against the area under the curve of GRAB-5-HT signal during consumption in both food (black dots) and water (white dots) restriction conditions. Dashed line indicates line of best fit for data points in the water restriction condition. ***D***, Preference index of the prefed flavor from both baseline and devaluation days are shown. ***E***, Difference score for the GRAB-5-HT signal during consumption of the pre-fed flavor relative to the control flavor is shown for both baseline and devaluation conditions. ***F***, GRAB-5-HT difference score is plotted against the change in preference from baseline to devaluation day.

We applied a similar transient-based analysis to GRAB-5-HT signals collected during the six-bottle variant of the paradigm to determine the effect of consumption at different concentrations on transient magnitude and duration. 5-HT transients were both longer (Fig. 3*E*) and of greater magnitude (Fig. 3*F*) when animals were consuming any concentration of milk (Kruskal–Wallis: *H*_Magnitude_(6, *N* = 2,073) = 174.31, *p* < 0.001; *H*_Duration_(6, *N* = 2,073) = 122.44, *p* < 0.001). Interestingly, transient duration (*W* = 640; *p*_holm _= 0.027), but not magnitude (*W* = 981; *p*_holm _= 0.27), was also significantly greater during water consumption relative to transients that occurred outside of licking. Therefore, DMS serotonin release was more likely to occur during reward consumption, and reward-associated release was quantitatively different than release that occurred both outside of consummatory periods and during consumption of lower-valued reward.

### Internal state influences serotonin encoding of reward

We were interested in whether DMS serotonin specifically encodes extrinsic reward value or if serotonin levels could also be influenced by internal state. In two experiments, we increased or decreased the subjective value of a reward to determine how the manipulation influenced the reward-related serotonin signal. We first measured the GRAB-5-HT signal during water consumption under water-restricted conditions to increase the subjective value of water. Since we were interested in the effect of internal state on serotonin signaling, mice that did not display increased water drinking under water deprivation conditions were initially excluded from the group comparison of the GRAB-5-HT signal. The GRAB-5-HT signal was analyzed for the mice that consumed more water under water restriction conditions (*n* = 8; interaction: *F*_(1,7)_ = 74.36; *p* < 0.001; *Ψ*_(7)Food _= 10.66; *p* < 0.001; Ψ_(7)Fluid _= 1.26, *p* = 0.25). As expected based on the results shown in Figure 2, there was significantly more 5-HT released during milk consumption compared with water (main effect of reward type: *F*_(1,7)_ = 10.17; *p* = 0.015; Fig. 4*A*). However, there was no significant main effect of deprivation state on 5-HT signal (*F*_(1,7)_ = 3.4; *p* = 0.10), nor a significant interaction between restriction state and reward type (*F*_(1,7)_ = 0.24; *p* = 0.64). Additionally, there was no significant increase in 5-HT release during water consumption during food- or water-restricted conditions (planned comparison between water-elicited GRAB-5-HT signal during food versus water restriction: *t*_(7)_ = 1.2726; *p* = 0.2438). However, given the suggestive but nonsignificant increase in GRAB-5-HT in the water-restricted mice, we followed up with a correlational analysis, including the mice that were originally excluded based on their lack of increased water consumption (Fig. 4*C*). There was a significant correlation between amount of water consumption and 5-HT release (*R*^2 ^= 0.345; *p* = 0.021). This suggests that when a reward is subjectively valued more, it is associated with a larger release of 5-HT in the DMS.

We next decreased the subjective value of a reward through satiety-induced devaluation of a flavored reward. The initial preference for banana or chocolate flavor was not significantly different at baseline, with mice equally preferring both flavors (*t*_(15)_ = 0.46; *p* = 0.65). Following devaluation of one of the flavors, the majority of mice (10 of 16) shifted their preference toward the nondevalued flavor (Fig. 4*D*); thus, all mice were included in the subsequent analysis of the GRAB-5-HT signal. Mice showed a significantly reduced GRAB-5-HT signal to the devalued reward compared with the valued reward (*t*_(15)_ = 2.66; *p* = 0.018; Fig. 4*E*). There was also a positive correlation between decreased preference for the devalued flavor and decreased 5-HT release during its consumption (Fig. 4*F*; *R*^2 ^= 0.411; *p* = 0.0075), suggesting that reward-related serotonin release is muted when the subjective value of the reward is decreased. Taken together with the water restriction data, these results show that serotonin in the DMS encodes the subjective value of rewards based on internal state.

### Striatal serotonin encodes reward anticipation, in addition to reward consumption

**Figure 5.**
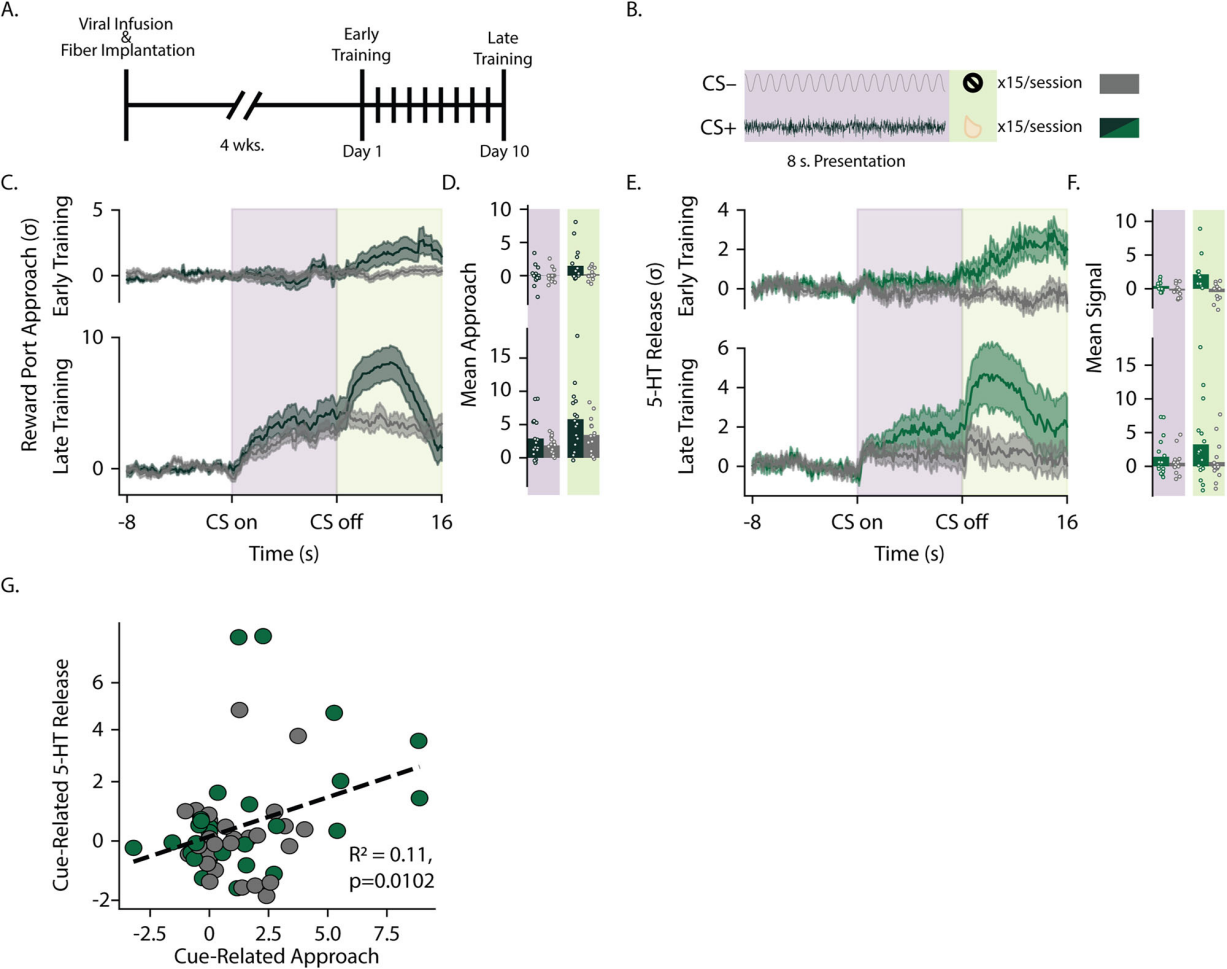
DMS serotonin increases during conditioned approach. ***A***, Animals were trained for 10 d in a pavlovian conditioning paradigm beginning 4 weeks after surgery with GRAB-5-HT signal recorded on Day 1 (early training) and Day 10 (late training). ***B***, The trial structure of the auditory pavlovian conditioned approach paradigm is shown. White noise and pure tones were presented as 8 s auditory cues before delivery of evaporated milk reward (CS+; green lines in all subsequent panels) or no reward (CS−; gray lines in all subsequent panels) and counterbalanced across animals. ***C***, Behavioral approach is shown as the time spent at the reward port during CS+ versus CS− presentations after training during early (top panels) and late (bottom panels) training. ***D***, Averages across cue and reward periods in early and late training are shown for individual animals (dots) and group averages (bars). ***E***, GRAB-5-HT signal is shown for CS+ (green) and CS− (gray) trials during early (top panels) and late (bottom panels) training. ***F***, AUCs for the GRAB-5-HT signal are shown for individual mice (dots) and as group averages (bars) during cue and reward periods in early and late training. ***G***, The association between behavioral approach is plotted against GRAB-5-HT signal during the cue period for each mouse's average across CS+ (green) and CS− (gray) trials during early and late training.

Mice were trained for 10 sessions in an appetitive pavlovian auditory conditioning paradigm, and we recorded the GRAB-5-HT signal during the first and last days of conditioning (Fig. 5*A*). An 8 s presentation of a tone or white noise preceded reward delivery (CS+) or the absence of reward (CS−; Fig. 5*B*). Over training, mice learned to approach the reward port more during the CS presentations (Fig. 5*C*, main effect of training *F*_(1,15)_ = 31.21; *p* < 0.001). Although there was some generalization across both cues, mice approached more during CS+ compared with the CS− trials (main effect of CS: *F*_(1,15)_ = 5.57; *p* = 0.032), and this effect increased across training (CS × training interaction: *F*_(1,15)_ = 5.29; *p* = 0.036; Fig. 5*D*). Controlling for behavioral differences, we analyzed the GRAB-5-HT signal and found significantly elevated 5-HT levels during the CS+ compared with the CS− trials (Fig. 5*E*; *F*_(1,83)_ = 14.95; *p* = 0.0002). Additionally, this was influenced by trial epoch (cue × epoch interaction: *F*_(1,83)_ = 5.10; *p* = 0.026), with greater 5-HT release during the reward period compared with the cue period for CS+ trials (*F*_(1,38)_ = 6.081; *p* = 0.018) but not CS− trials (*F*_(1,38)_ = 0.06; *p* = 0.802). There was a suggestive, but not significant difference between the 5-HT levels during the cue period of CS+ and CS− trials (*F*_(1,38)_ = 2.80; *p* = 0.10). Given the individual variability in both the behavior and GRAB-5-HT signal during the cue period, we looked to see if there was a correlation between the 5-HT signal and the reward port approach during the cue period. There was a significant correlation between serotonin and approach behavior during the cue period of the trial (Fig. 5*G*; *R*^2^ = 0.112; *p* = 0.01), suggesting that serotonin can track how much a mouse anticipates an upcoming reward. Overall, these data show that striatal serotonin encodes some aspect of reward anticipation or expectation that is independent of reward consumption.

## Discussion

Understanding how serotonin modulates the neural circuitry of reward processing has important implications for the many treatment approaches in psychiatry which target serotonin signaling. Given the previous lack of available techniques to measure serotonin release on a time scale compatible with resolving the encoding of individual cues and rewards, there is little known about the temporal characteristics of serotonin release in this context. We used a serotonin biosensor (GRAB-5-HT) in mice to identify the timing and characteristics of the release of serotonin in vivo in the DMS in relation to rewards, reward value, and cued rewards. Overall, our results showed that DMS serotonin begans to rise ∼2 s before the consumption of a reward, and peaked around the time of consumption. Transient analyses of the GRAB-5-HT signal showed that serotonin was more likely to be released during reward consumption, and the release that occurred during reward consumption was likely to be larger. Additionally, serotonin release was graded based on reward value, with the highest reward concentrations eliciting the highest magnitude and most prolonged serotonin signal. Intrinsic reward value also influenced serotonin release, with serotonin levels tracking both increases and decreases in subjective value. Finally, in a pavlovian conditioning experiment, we showed that 5-HT encodes reward anticipation, though to a lesser extent than reward consumption. Overall, serotonin encodes external reward value in the DMS and may be important for promoting reward approach.

We were surprised that the value-coding feature of serotonin release in the DMS was stronger for external value and only weakly modulated by our manipulations to internal state-driven changes to subjective value. This was seen in relatively small changes in 5-HT release to water when mice were subject to water restriction. Additionally, despite the significant decrease in preference for milk when mice were given access to both solutions under water restriction, mice still preferred milk to water, suggesting that consuming milk remained of greater value than consuming water. A possible explanation for this is that serotonin may be involved in the DMS representation of action value, in which a less-preferred option is encoded as such even in fixed-choice scenarios (Ito and Doya, 2015). Therefore, it is possible that a larger effect of internal state on DMS serotonin release exists but that 24 h of fluid deprivation did not change subjective value enough to see such an effect commensurate with the changes in external reward value. To this point, satiety-induced devaluation of a flavored reward resulted in decreased serotonin release to the devalued flavor suggesting that serotonin can track preference. This is consistent with the finding that DRN inhibition impairs the ability of animals to perform such devaluation (Ohmura et al., 2021) and further suggests that the DMS is a possible site of action for serotonin to mediate this behavior (McDannald, 2021). While we found that serotonin release was variably modulated by internal state, this contrasts with findings showing that dopamine release is tightly linked to homeostatic signals of thirst and hunger and strongly modulated by these internal states (Cone et al., 2014; Hsu et al., 2020).

While there are some similarities between our results measuring serotonin release and previous published reports of dopamine release in the DMS in the context of reward, there are additional notable differences, particularly in the encoding of anticipation. Serotonin and dopamine are similarly both released in response to reward predictive cues (Brown et al., 2011; Li et al., 2016; Matias et al., 2017). However, while dopamine does encode unexpected rewards in other brain regions, such as the ventral striatum, dopamine release in the DMS predominantly occurs in response to reward-predictive cues following associative conditioning (Brown et al., 2011). This contrasts with the high levels of serotonin release in the DMS that we report in response to unexpected rewards in the absence of conditioning. Another difference includes the time scales of the dopamine and 5-HT signals. Dopamine signals seem to show a faster onset and clearance (Brown et al., 2011), while serotonin signals are longer. In our results, 5-HT release is prolonged throughout the duration of the 8 s predictive cue. This is also consistent with past reports using electrophysiology and calcium imaging in the DRN (Zhong et al., 2017). Dopamine release, however, occurs phasically at the onset of the predictive cue (Brown et al., 2011). While the different time courses are difficult to compare quantitatively given the differences in the kinetics of the binding and fluorescence of the 5-HT and dopamine biosensors used to measure release, the subsecond off-kinetics of the GRAB-5-HT sensor make it unlikely that the prolonged 5-HT signal over 8 s is only due to biosensor lag (Deng et al., 2024).

The different timescales of dopamine and serotonin release leads to theories that these signals work together to allow flexible behavior under conditions of uncertainty. Serotonin has been proposed to be released under conditions of uncertainty to inhibit extraneous inputs and facilitate targeted excitation by dopamine, essentially boosting the signal-to-noise ratio of dopamine reward signals (Matias et al., 2017). While this is consistent with electrophysiological work in the ventral striatum that finds dopamine and serotonin to filter excitatory inputs (Christoffel et al., 2021), other work that finds serotonergic DRN neurons to be less active during uncertainty suggest greater complexity of function (Grossman et al., 2022). Future work that directly assesses the effect of uncertainty in the environment on both dopamine and serotonin in the DMS will be necessary to clarify their precise function. It may be that serotonin is engaged by surprise and uncertainty to facilitate labile behavior and promote state-switching (Matias et al., 2017; Odland et al., 2021). An alternate hypothesis would propose that serotonin is elevated when a beneficial outcome is expected and enables persistence toward it (K. W. Miyazaki et al., 2014; K. Miyazaki et al., 2020; Grossman et al., 2022). Our data mostly supports this latter idea, given that we found evidence for an anticipatory signal associated with reward approach but that the reward consumption-related signal is largely unchanged by expectation.

Our results reporting serotonin release in an associative learning paradigm are consistent with some prior studies measuring somatic activity of the DRN (Li et al., 2016; Zhong et al., 2017), though not all (Matias et al., 2017). In some results, somatic activity does not decay as a reward becomes predicted (Li et al., 2016; Zhong et al., 2017), though others have found that expectation influenced 5-HT encoding of outcome (Matias et al., 2017). It has been proposed that the previously seen persistence of reward associated 5-HT after learning may be a feature of uncontrollable events, such as intraoral delivery of reward (Li et al., 2016; Zhong et al., 2017) or forced exposure to an aversive air puff (Matias et al., 2017). However, we observed persistent reward encoding in a design where reward was voluntarily retrieved. It is possible, then, that a subset of DRN neurons which do scale back their activity with learning do not project to the DMS, given that the serotonergic neurons in the DRN are heterogeneous and can be functionally segregated by projection pathway (Ren et al., 2018; Paquelet et al., 2022).

Overall, our results show that serotonin release occurs during multiple phases of reward processing—including anticipation, approach, and consumption. Additionally, we found that DMS serotonin encodes reward value, seen through graded responses to different concentrations of reward and changes in subjective value. This aligns with previously observed patterns of somatic activity among SERT+ neurons in the DRN including a graded response to concentrations of reward (Paquelet et al., 2022) and a strong response to surprising outcomes (Matias et al., 2017). We report that DMS serotonin release anticipates reward during tasks that require approach to receive the reward and neutral stimuli are encoded only after they have been repeatedly associated with reward. Our data are consistent with previously described theories of DRN/5-HT function, particularly in representing subjective well-being and/or the value of persistence and anticipated outcomes (M. Luo et al., 2016; Doya et al., 2021; Ohmura et al., 2021; Q. Luo et al., 2023). Beyond classical conditioning, monitoring 5-HT during other DMS-dependent tasks will help to clarify how serotonin may contribute to the acquisition and execution of action–outcome contingencies. In sum, this research advances our understanding of 5-HT function in the DMS and provides insight into the representation of reward value—intrinsic, extrinsic, and anticipated—which informs how striatal serotonin modulates reward-related behaviors.
